# Identification of Mycobacterium tuberculosis transcriptional repressor EthR inhibitors: Shape-based search and machine learning studies

**DOI:** 10.1016/j.heliyon.2024.e26802

**Published:** 2024-02-22

**Authors:** Rupesh V. Chikhale, Gaber E. Eldesoky, Mahima Sudhir Kolpe, Vikramsinh Sardarsinh Suryawanshi, Pritee Chunarkar Patil, Shovonlal Bhowmick

**Affiliations:** a Department of Pharmaceutical and Biological Chemistry, School of Pharmacy, University College London, London, UK; b Chemistry Department, College of Science, King Saud University, Riyadh, 11451, Saudi Arabia; c SilicoScientia Private Limited, Nagananda Commercial Complex, No. 07/3, 15/1, 18th Main Road, Jayanagar 9th Block, Bengaluru, 560041, India; d Department of Bioinformatics, Rajiv Gandhi Institute of IT and Biotechnology, Bharati Vidyapeeth Deemed to be University, Pune-Satara Road, Pune, India

**Keywords:** Ethionamide, EthA gene repressor, Transcriptional repressor, ADMET, Drug resistance

## Abstract

Tuberculosis has been a challenge to the world since prehistoric times, and with the advent of drug-resistant strains, it has become more challenging to treat this infection. Ethionamide (ETH), a second-line drug, acts as a prodrug and targets mycolic acid synthesis by targeting the enoyl-acyl carrier protein reductase (InhA) enzyme. *Mycobacterium tuberculosis* (Mtb) EthR is an ethA gene repressor required to activate prodrug ETH. Recent studies suggest targeting the EthR could lead to newer drug molecules that would help better activate the ETH or complement this process. In this report, we have attempted and successfully identified three new molecules from the drug repurposing library that can target EthR protein and function as ETH boosters. These molecules were obtained after rigorous filtering of the database for their physicochemical, toxicological properties and safety. The molecular docking, molecular dynamics simulations and binding energy studies yielded three compounds, Ethyl (2-amino-4-((4-fluorobenzyl)amino)phenyl)carbamate) (L1), 2-((2,2-Difluorobenzo [d] [1,3]dioxol-5-yl)amino)-2-oxoethyl (E)-3-(5-bromofuran-2-yl)acrylate (L2), and N-(2,3-Dihydrobenzo [b] [1,4]dioxin-6-yl)-4-(2-((4-fluorophenyl)amino)-2-oxoethoxy)-3-methoxy benzamide (L3) are potential EthR inhibitors. We applied machine learning methods to evaluate these molecules for toxicity and synthesisability, suggesting safety and ease of synthesis for these molecules. These molecules are known for other pharmacological activities and can be repurposed faster as adjuvant therapy for tuberculosis.

## 1 Introduction

Tuberculosis (Tb) is an infectious disease caused by the *Mycobacterium tuberculosis* (Mtb), an aerobic, pathogenic bacteria from the Mycobacteriaceae family [1]. According to one school of thought, Mtb co-evolved with humans, and it travelled from Africa to various parts of the globe with the human settlements, making them one of the oldest pathogens infecting prehistoric and modern humans [2]. Tb is endemic to most parts of the world with more prevalent in developing countries compared to developed nations, however, illegal immigration and thus lack of treatment and diagnosis is becoming one of the reasons for its spread through the globe [3]. According to the data from the World Health Organisation (WHO), there were about 1.6 million deaths due to Tb in the year 2021 including 187,000 coinfected with HIV. It was also estimated that 10.6 million people were infected with Tb including 1.2 million children in 2021 [4]. The rise in Multi Drug Resistant (MDR) is one of the most concerning facts reflected in the Global Tuberculosis Report 2022, which suggests an increase in cases during 2015–21 [5,6]. The drug-sensitive Tb can be treated with first-line drug therapy for 6 months, which includes a combination of Isoniazid, Rifampicin, Pyrazinamide and Ethambutol. The second-line drugs are used in different combinations depending on the drug sensitivity and the patient response, these drugs are Streptomycin, Kanamycin, Amikacin, Capreomycin, Ofloxacin, Ethionamide, Terizidone and certain other antibiotics [7]. Linezolid, Bedaquiline and Delamanid are some of the drugs used in the treatment of MDR and extremely drug-resistant (XDR) tuberculosis [8]. The development of drug resistance in Tb is at various levels, these can be defined as mono-resistance (one of the first-line *anti*-Tb drugs), Poly-resistance (resistance to isoniazid, rifampicin and one or more first-line drugs), MDR (resistance to isoniazid and rifampicin), XDR (resistance to fluoroquinolone + one of the 3-s line injectable drug, capreomycin, kanamycin, amikacin) and Rifampicin resistance (RR) [9]. The treatment for these forms of Tb varies froam a minimum of six months to more than two years, depending on the drug response, patient compatibility, and socioeconomic conditions [10]. The global disease burden, lengthy duration of therapy and the increasing occurrence of drug resistance have challenged the scientific community to urgent need for novel *anti*-Tb drug molecules. There are various approaches taken to answer this challenge, like the development of the Tb vaccine [11], new drug molecules [12], investigation of new drug targets in Mtb [13] and revisiting the old target with innovative approaches [[14], [15], [16]]. Some Tb drugs, such as ethionamide (ETH) and isoniazid (INH), are structurally analogous and need to be activated before performing their desired biological activity. In this case, both drugs target enoyl-acyl carrier protein reductase (InhA), which is an enzyme necessary for mycolic acid synthesis [17]. ETH is an important second-line drug used in treating Tb, and the mechanism of action is very well understood for ETH. Besra and Baulard [18], explained the ETH activator Rv3854c and termed it EthA, it is analogous to the various monooxygenases and induces ETH sensitivity on overexpression in Mycobacteria. Later, Montellano and coworkers established the EtaA as a FAD-containing enzyme that oxidizes ETA to its corresponding S-oxide. This is further oxidized to 2-ethyl-4-amidopyridine via the formation of intermediate sulfinic acid. Mtb EthR is an ethA gene repressor required to activate prodrug ETH. EthR is a homodimer with a helical structure and shows similarity to the TetR family members [19].

**Fig. 1:**
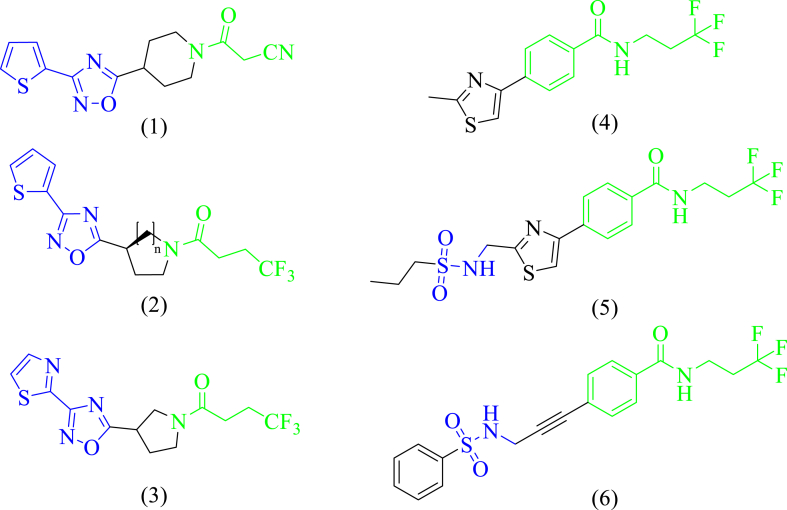
Recent inhibitors of Mtb transcriptional repressor EthR and the scaffold similarities in them: (**1**) BDM31343; (2) Thiophen-2-yl-1,2,4-oxadiazoles; (3) BDM41906, (**4**) 4-(2-Methylthiazol-4-yl)-N-(3,3,3-trifluoropropyl) benzamide; (**5**) 4-(2-(Propylsulfonamidomethyl)thiazol-4-yl)-N-(3,3,3-trifluoropropyl) benzamide; (**6**) 4-(3-(Phenylsulfonamido)prop-1-ynyl)-N-(3,3,3-trifluoropropyl) benzamide (**L5**).

Baulard and coworkers [20] produced a novel approach of inhibiting the EthR by small molecule inhibitors and thus boosting the bioactivation of ETH, one of the lead molecules from this study was BDM31343 (**1**) (Fig. 1 (1)). Later, they reported the synthesis, biological studies and SAR for a series of thiophen-2-yl-1,2,4-oxadiazoles as inhibitors of EthR (**2**) (Fig. 1 (2)) with improved activity and some physicochemical properties like solubility and metabolic stability [21]. These ligands were further optimized in the next attempt and BDM41906 (**3**) (Fig. 1 (3)) [22] was obtained because of structure-based drug design. This molecule had improved efficacy and better pharmacokinetic properties. They also applied an alternative approach of fragment-based design using a mix of surface plasmon resonance assay (SPR), X-ray crystallography, and SAR, leading to the identification of a potent molecule, 4-(2-Methylthiazol-4-yl)-N-(3,3,3-trifluoropropyl)benzamide (**4**, PDB: 4M3D) (Fig. 1 (4)) with IC_50_ = 0.40 μM, EC_50_ = 0.08 μM and high ligand efficiency of LE = 0.47, it also shows good solubility [23]. In this series other molecules that we found interesting were 4-(2-(Propylsulfonamidomethyl)thiazol-4-yl)-N-(3,3,3-trifluoropropyl)benzamide (**5**, PDB: 4M3E) (Fig. 1 (5)) and 4-(3-(Phenylsulfonamido)prop-1-ynyl)-N-(3,3,3-trifluoropropyl)-benzamide (**6**, PDB: 4M3D) (Fig. 1 (6)), these compounds show similar interactions as the best compound from the series when observed from their cocrystal structures. The concept of identification of new inhibitors of Mtb transcriptional repressor EthR is very logical and highly attractive. Herein, we are reporting the application of highly advanced tools in structure-based drug discovery methods for identifying novel molecules from large, small-molecule databases. Herein, the focus lies on structural similarities within the chemical scaffolds with known EthR inhibitors, as discussed earlier in Fig. 1, and how these could be explored to mine the ligands from drug repurposing databases.

## 2 Materials and methods

### 2.1 Selection of a large chemical library database and initial curation of the database for screening

A large compound pool of ∼160,383 small molecules was downloaded from Selleckchem [24] (https://www.selleckchem.com/), a top-rated chemical supplier across the globe containing a diverse set of chemicals ready to use for virtual screening purposes. Moreover, it has been stated that more than 120,000 compounds are associated with being used to research cell signalling pathways. As one of the top chemical suppliers of the newest inhibitors, Selleckchem actively follows the most recent scientific developments to benefit pharmaceutical industries or other research projects. Over ∼8000 small compounds in Selleck's Bioactive Compound Libraries have been shown to have biological and pharmacological properties. Moreover, the selected database includes compounds entered in preclinical and clinical studies and have proven their efficacy and safety, and several of them have received FDA approval. To begin with, three major filtration criteria, Lipinski's rule of five (Ro5), Veber's rule and Drug-likeness properties were used to screen the database and further curated for retaining most potential drug-like candidates. Such properties screening was implemented in Python RDKit (https://www.rdkit.org). Besides the large Selleckchem chemical library, a small set of 30 *anti*-Tb compounds were separately collected. These 30 TB drugs were used in the study setting to compare and validate the study outcome.

### 2.2 Preparation of the curated chemical library dataset and FDA-approved TB drugs

The ligand molecules were obtained following earlier mentioned filtration criteria, and the 30 *anti*-Tb drugs were prepared according to the standard protocol using the OpenBabel tool [25]. In the ligand preparation step, the 3D conformation of the compounds was generated, and hydrogen (-H) atoms were added. The pH and partial charge for the ligands in the database were adjusted where needed. Subsequently, these were converted into ‘pdbqt’ file format which is necessary for the AutoDock Vina docking tool [26]. In particular, the “PDBQT” file contains the information on Partial Charge (Q) & Atom Type (T) for a specific ligand. Displaying the normal PDB files does not hold such information. In contrast, PDBQT files constitute an additional column that lists the partial charge (Q) and the assigned AutoDock atom type (T) for each atom in the molecule. The AutoDock atom types provide a better insight toward granular differentiation between atoms, such as listing aliphatic and aromatic carbons as separate AutoDock atom types.

### 2.3 Selection of target protein and preparation

The 3D coordinates of the *Mtb* HTH-type transcriptional regulator EthR crystal structure (PDB ID: 4M3D [27]) were obtained from the RCSB-Protein Data Bank (PDB) [28]. The EthR crystal structure (PDB ID: 4M3D) shows resolution and R-value of 1.90 Å and 0.22, respectively. The selected *Mtb* EthR consists of 216 amino acid residues with no mutation. The desired protein was prepared using AutoDockTools (ADT) [29] by adding H-atoms and removing all hetero atoms, including water molecules. Moreover, Kollman charges were added to the protein to adjust the convenient partial charges to the selected Mtb protein. Finally, the prepared protein was saved into the desired file format like ‘pdbqt’ for later analysis. Moreover, the preferred protein of interest also consists of the attached EthR inhibitor, 4-{3-[(phenylsulfonyl)amino] prop-1-yn-1-yl}-N-(3,3,3-trifluoropropyl) benzamide, which was also prepared as mentioned under ligand preparation.

### 2.4 Molecular docking

All prepared ligands and the EthR protein were allowed for molecular docking study using the MPI Vina platform [30] installed on the Linux operating system. However, before submission of the docking job, the configuration file for AutoDock Vina was created by keeping the required set of information like the protein's centre grid box coordinates along x, y, and z-axes. The rest of the parameters for molecular docking were kept as default in the configuration file. All the compounds of the Selleckchem database kept from the previous step, and 30 FDA-approved TB drugs and co-crystal ligands were docked with *Mtb* EthR employing standard docking protocol. After successfully executing docking for all compounds said above, their binding affinity was critically checked to further reduce the chemical space for better selection of Mtb EthR-selective compounds. Therefore, all 30 FDA drugs and co-crystal ligand binding affinity score and interaction mode were critically checked to set the threshold binding affinity score for Selleckchem compound database screening and further analysis. The lowest binding affinity score of 30 FDA drugs and co-crystal ligands were compared, and the lowest binding affinity score was considered as a threshold for subsequent analysis for the selection of potential compounds. Moreover, the binding interaction mode was explored using PLIP–a web-based protein-ligand interaction retrieval program [31].

Furthermore, it is crucial to note that any molecular docking method must first be validated before being used in the screening of any chemical compound datasets. It was always predicted that molecular docking will result in a conformational orientation that resembles the conformation of a ligand linked to a co-crystal. Hence, self-docking is one of methods for verifying the molecular docking process. This method verifies the co-crystal ligand's binding mode or orientation. In particular, the co-crystal ligand that was initially bound was re-docked to the active binding site using same docking grid parameters used for rest of compounds datasets.

### 2.5 Post-docking processing of compounds and subsequent ADMET based filtration using pkCSM

The pkCSM [32] web-tool (https://biosig.lab.uq.edu.au/pkcsm/prediction) was used for ADMET (absorption, distribution, metabolism, excretion, and toxicity) properties-based filtration on best hit compounds retained from molecular docking analysis. ADMET is an important criterion here implemented to reduce the chances of selecting the compounds with adverse side effects and thereby also reducing the failure rate for compounds’ efficacy and safety deficiencies. *In-silico* ADMET prediction approach has long been used as an important parameter in the pharmaceutical or drug discovery pipeline for reducing the risk of late-stage attrition and helping in the choice of potentially good compounds from large compound datasets or optimization of new chemical entities. For the execution of ADMET in pkCSM, all best hits (∼3194) compounds obtained from docking study were browsed in standard SMILE file format and prediction of all pharmacokinetic properties was estimated under ADMET.

### 2.6 Machine learning based toxicity and synthetic accessibility assessment

Machine learning (ML) is widely used to explore the toxicity and other drug-likeness assessment of small molecules [33]. Molecules found after the screening of SelleckChem against Mtb protein were taken for toxicity and synthetic accessibility calculation using eToxPred tool [34]. It uses ML models to predict the toxicity of the given molecules directly from the molecular fingerprints of chemical compounds. The models in eToxPred are trained and confirmed through several diverse datasets including known drugs, potentially hazardous chemicals, natural products, and synthetic bioactive compounds.

Machine learning models in eToxPred are trained and cross-validated against a number of datasets comprising known drugs, potentially hazardous chemicals, natural products, and synthetic bioactive compounds. The models are developed by the Extra Tree (ET) ML algorithm. Along with the ET, another three ML algorithms such as Gradient Boosting (GB), Balanced Random Forest (BRF) and Balanced Bagging (Confirmers to develop the ML models with their given datasets [35,36]. Prior to use in the prediction of toxicity and SA scores, all the developed models were validated through the calculation of receiver operating curve (ROC) area under the curve (AUC) and accuracy. The validated models were used to calculate the overall toxicity value which suggests whether the molecule is toxic or non-toxic in nature. The toxicity value ranges from 0 to 1 showing non-toxic to toxic, respectively. Along with toxicity, the eToxPred also gives the synthetic accessibility (SA) of any given molecule. The SA score represented 1 to 10 signifying easy to difficult in synthesis, respectively.

### 2.7 Molecular dynamics (MD) simulations analysis of the protein-ligand complex and MM-GBSA

Through all atomic MD simulation analyses, the dynamic nature of the selected screened four protein-ligand complexes, including the co-crystal and best standard TB drug compounds bound with EthR complexes, was assessed. The MD simulation approach helps to understand the inter- or intra-molecular binding interactions between protein-ligand macromolecular complexes and their reorganisation behaviour in different energetically induced conformational states in a dynamic environment. In this study total of six protein-ligand complexes were subjected for 100 ns all atomistic classical MD simulations. The MD simulation run was conducted with a time step of 2 fs, at a constant pressure of 1 atm, and constant temperature of 300 K. The *Mtb* EthR protein topology was produced using the CHARMM36 all-atoms forcefield, while SwissParam [37], an open-source server available online was used to create the topology of the final small molecules under consideration for MD simulation. Each protein-ligand complex system was solvated using the TIP3P water model and submerged into a cubic box before the simulation run. To neutralise the bio-macromolecular system as a whole, the suitable numbers of Na^+^ and Cl^−^ were adjusted. Each protein-ligand system underwent 50,000 steps of the steepest descents energy minimization approach to eliminate steric overlap. The SHAKE method was used to restrict the hydrogen atoms. The long-range electrostatic interactions were evaluated, implying the Particle Mesh Ewald (PME) [38] method and the cut-off value for non-bonded interactions was established at the cut-off value of 8 Å. Langevin thermostat with a 2.0 collision frequency was used to regulate the entire simulated system's overall temperature. At 1 atm Monte Carlo barostat with volume exchanges occurring every 100 fs was also used to regulate the system's pressure. NVT and NPT ensembles were used to perform rounds of the system equilibration, before acquiring the MD simulation data. A variety of different important analytic metrics, including protein backbone root-mean-square deviation (RMSD), ligand RMSD root-mean-square fluctuation (RMSF), and radius of gyration (RoG), were estimated following the successful completion of the MD manufacturing run during the 100 ns MD simulation span employing several GROMACS [39] integrated utility tools.

Additionally, each MD simulated protein-ligand complex's binding free energy (ΔG_*bind*_) was determined using the Generalised Born and Surface Area Solvation (MM/GBSA) method, employing the GROMACS embedded gmx_mmpbsa utility package [40]. The complete set of 10,000 frames was considered for the binding free energy calculation with an interval of 5. The detailed mathematical expression was considered to determine ΔG_*bind*_ has discussed previously [41,42], elsewhere.

## 3 Results and discussion

### 3.1 Screening of SelleckChem database against Mtb

**Fig. 2:**
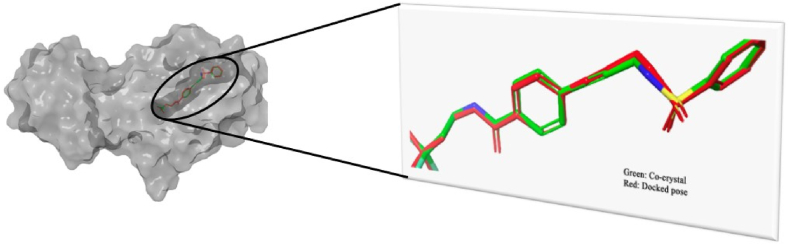
Superimposed structure of co-crystal conformation and best docked pose of the same.

The Selleckchem database consisting of 160,383 ligands was prepared and were filtered for their physicochemical properties and drug-likeness. Herein, we considered Lipinski's rule of five, Veber's rule and Drug-likeness as filters to narrow down the chemical space for further evaluation. This first pass led to a total of 119,854 molecules retained from the database. These molecules were subjected to molecular docking studies using AutoDock Vina running in MPI mode where the active site of the Mtb transcriptional repressor EthR was targeted. Along with the Selleckchem database we have also considered 30 *anti*-TB drug molecules for the docking studies. The protein crystal structure was retrieved from the RCSB-PDB and was prepared for docking experiment. The native ligand bound to the crystal structure of EthR was extracted and used in the docking studies as a control. To validate the docking protocol, the self-docking approach was used in which the structure of the bound co-crystal ligand was re-drawn and docked at the active site. The best-docked pose and co-crystal ligand were superimposed and RMSD was calculated. Multiple docking of the re-drawn co-crystal ligand was performed and superimposed to record the RMSD. The best protocol with superimposition RMSD of 0.182 Å was identified and used for the further screening of the Selleckchem database. The superimposed co-crystal conformation and best docked pose is given in Fig. 2.

**Fig. 3:**
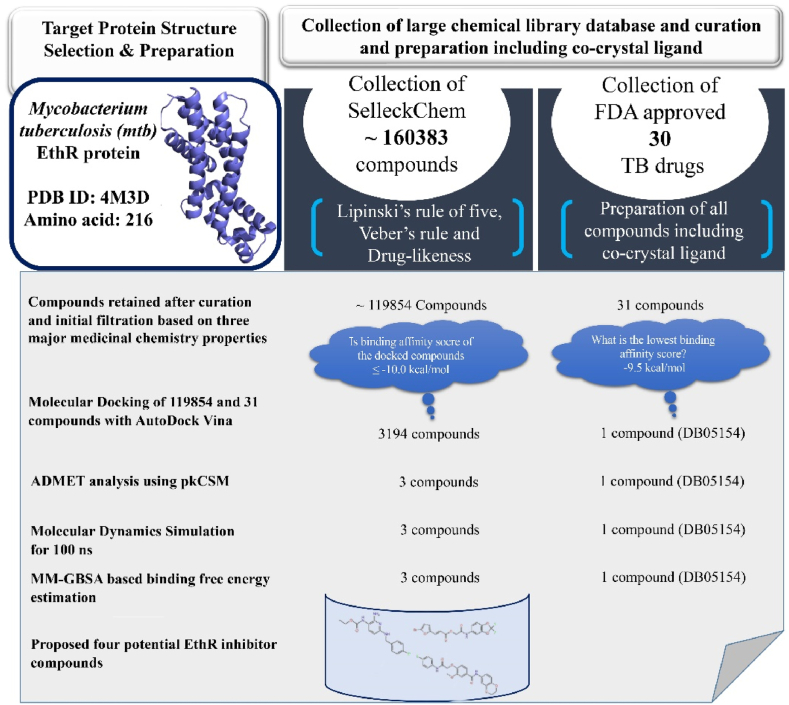
Virtual screening workflow for the screening of the SelleckChem database against Mtb.

The main aim of the current piece of work was to find some potential lead-like molecules for therapeutic application in Tb infection through the virtual screening approach. The detailed workflow of the work is given in Fig. 3.

**Fig. 4:**
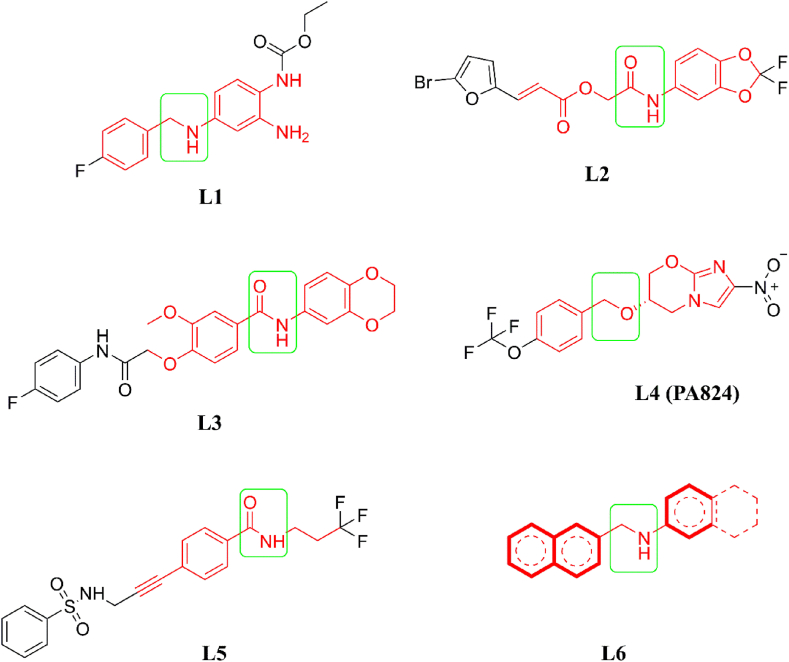
The final selected molecules from molecular docking experiments: **L1** (Ethyl (2-amino-4-((4-fluorobenzyl)amino)phenyl)carbamate), **L2** (2-((2,2-Difluorobenzo [d] [1,3]dioxol-5-yl)amino)-2-oxoethyl (E)-3-(5-bromofuran-2-yl)acrylate), and **L3** (N-(2,3-Dihydrobenzo [b] [1,4]dioxin-6-yl)-4-(2-((4-fluorophenyl)amino)-2-oxoethoxy)-3-methoxybenzamide) were obtained as hit ligands from the Selleckchem database following molecular docking. **L4**, also known as Pretomanid ((*S*)-2-Nitro-6-((4-(trifluoromethoxy)benzyl)oxy)-6,7-dihydro-5H-imidazo [2,1-b] [1,3]oxazine) is potent antimycobacterial compound and **L5** (4-{3-[(phenylsulfonyl)amino] prop-1-yn-1-yl}-N-(3,3,3-trifluoropropyl)benzamide) is the co-crystallised ligand from the crystal structure of EthR (PDB: 4M3D). **L6** represents common structural features for this series of EthR inhibitors.

On successful completion of the molecular docking, the binding energy of each molecule was extracted and analyzed. To reduce the chemical space to select better affinity molecules, a threshold of −10.0 kcal/mol was applied. By applying the above threshold in the binding energy, a total of 3194 molecules were found to have a binding energy of −10.0 kcal/mol or less. It is important to note that the co-crystal molecule was found to show a binding affinity of −9.5 kcal/mol. The above remained molecules were subjected to ADMET screening analysis using the pkCSM tool to obtain molecules having an acceptable pharmacokinetic profile as well as safe for the treatment of Tb. In particular, a number of pharmacokinetics and toxicity parameters were recorded and analyzed. Molecules were found to have intestinal absorption of less than 30, skin permeability of greater than −2.5, BBB of greater than −1, and CNS permeability greater than −3 were removed due to their poor pharmacokinetic profile. Molecules retained after the pharmacokinetic assessment were further taken into consideration for the toxicity assessment. For the above remaining molecules, AMES toxicity, hepatotoxicity and skin sensitivity were checked and compounds that showed negative response were taken for minnow toxicity and maximum tolerated dose analyses. It is recommended that molecules should have a minnow toxicity greater than −0.3 and a maximum tolerated dose less than 0.477. By following the above criteria, a total of 3 molecules were retained. The two-dimensional (2D) representation of the final selected molecules along with co-crystal bound ligand and best docked drug molecule is given in Fig. 4. For our convention and easy explanation, from here onwards, the above molecules are named **L1** (CAS ID: 150,812-12-7), **L2** (CAS ID: 807,608-56-6), **L3** (CAS ID: 734,545-13-2), **L4** (TB drug, Pretomanid, CAS ID: 187,235-37-6) and **L5** (Co-crystallised ligand, CAS ID: 1589819-46-4). It is important to note that all three selected molecules possess a common structural feature (**L6**) that is shown in a rectangular box in Fig. 4. On close observation of each of the selected molecules, it can be prominently visible a number of pharmacophoric features in terms of hydrogen bond (HB) acceptors and donors, ring aromatic, and hydrophobic. Therefore, it is highly expected that selected molecules will form several binding interactions with the active site amino acids in the forms of HB, hydrophobic, and π-staking. those will help to hold the molecules in the binding site to exhibit the desired effects.

### 3.2 Pharmacokinetic, toxicity and synthetic accessibility

The pharmacokinetic and toxicity parameters for the final three molecules were predicted from the pKCSM and these are given in Table 1. The molecules' molecular weight and logP are less than 500 kDa and 5.0, respectively. The rotatable bonds were found to be 5, 5 and 7 for **L1**, **L2** and **L3** (Fig. 4), respectively, which suggest molecules are not rigid and are highly flexible. Other pharmacokinetic parameters such as intestinal absorption, skin, BBB and CNS permeability were in acceptable range. No toxicity parameters such as AMES, maximum tolerated dose, hepatoxicity, skin sensitivity and minnow toxicity were found in the toxic range. Hence, from the acceptable pharmacokinetic and toxicity profile, all molecules will be safe and easy to reach at the target side.

**Table 1:** Pharmacokinetic and toxicity assessment of final selected molecules.

	aMW	LogP	bNRB	cIA	dSP	eBBB	fCNS	AMES	gMTD	hHT	iSS	jMT
**L1**	304.325	2.983	5	87.818	−2.864	−1.259	−3.281	No	0.032	No	No	0.926
**L2**	429.856	3.347	5	94.469	−2.760	−0.988	−3.190	No	0.540	No	No	−1.469
**L3**	452.438	3.875	7	99.833	−2.748	−0.428	−3.114	No	0.398	No	No	−0.905

a Molecular weight.

b Number of rotatable bonds.

c Intestinal absorption.

d Skin permeability.

e Blood brain barrier.

f Central nervous system.

g Maximum tolerable dose.

h Heptotoxicity.

i Skin sensitivity.

j Minnow toxicity.

Further, the toxicity and synthetic accessibility of the molecules were predicted through the ML-based eToxPred tool. For the prediction, the default models of the tool developed using ET was used. In addition, three more models were developed using the same dataset using GB, BRF and BB algorithms. All four developed models were validated and found to show high accuracy. The ROC AUC of ET, GB, BRF and BB was found to be 0.970, 0.812, 0.962 and 0.917, respectively. The accuracy of each model was calculated, and it was found to be 0.884, 0.741, 0.875 and 0.829 for ET, GB, BRF and BB, respectively. The above data undoubtedly explained the high predictiveness of each model and can be used to predict any unknown molecule.

All four ML models were used to predict the selected model's toxicity and SA scores, which is given in Table 2. A toxicity score of less than 0.50 can be considered non-toxic. Not a single molecule was found to have a toxicity value of more than 0.430 when predicted using all four ML models. The above observation indicated that all molecules were non-toxic, corroborating the data found using the pkCSM tool. Further, the eToxPred was used to predict the SA score, which is given in Table 2. Molecules having a SA score of 5 or less can be easily synthesizable. All molecules were found to have an SA score of less than 5. Hence, all molecules are feasible to synthesize. It is also indicated that among three molecules, **L2** can most easily be synthesised followed by **L3** and **L1** (Fig. 4).

**Table 2:** Toxicity and SA score of selected molecules predicted through ML models.

Mols	Gradient Boosting	Extra trees	Balanced RF	Balanced Bagging	SAS
	TS				
**L1**	0.428	0.275	0.292	0.210	4.06
**L2**	0.181	0.244	0.249	0.000	0.63
**L3**	0.234	0.164	0.207	0.000	3.60

TS: Toxicity score; SAS: Synthetic accessibility score.

### 3.3 Binding interactions and MD simulation analysis

**Fig. 5:**
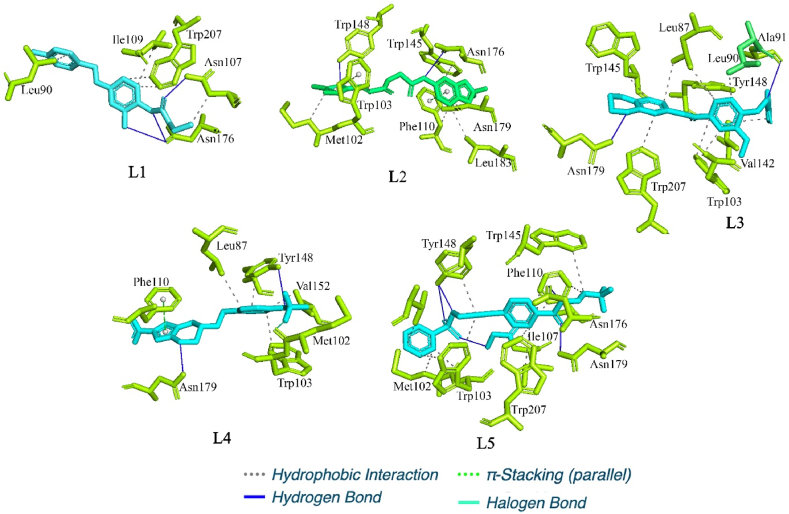
Molecular docking studies on the crystal structure of EthR, protein-ligand interactions between the three top hits **L1**, **L2** and **L3** from the docking studies, docked complex of the standard molecule pretomanid and the docked complex of the native co-crystal ligand.

The binding interaction profile of each of the three selected molecules (**L1**, **L2** and **L3**) along with **L4** and **L5** was explored, and it is given in Fig. 5 (**L1** – **L5**). The binding energy and CAS ID are given in Table 3. L1, **L2**, and L3 were shown to have binding energies of −12.45, −10.21, and −11.32 kcal/mol, respectively. In addition to the above, **L4** and **L5** were firmly bound with Mtb protein with the binding energy of −9.31 and −9.46 kcal/mol, respectively. It is seen that selected molecules from SelleckChem showed better binding affinity towards Mtb than **L4** and **L5**.

**Table 3:** Results from the molecular docking studies and MM-GBSA calculations.

Ligand	CAS no.	Dock Score	MM-GBSA (ΔG Kcal/mol)
**L1**	150,812-12-7	−12.45	−36.68
**L2**	807,608-56-6	−10.21	−32.57
**L3**	734,545-13-2	−11.32	−36.17
**L4**	187,235-37-6	−9.31	−28.34
**L5**	1589819-46-4	−9.46	−21.78

The binding site of **L5** consists of residues Lue87, Met102, Trp103, Gly106, Ile107, Phe110, Phe114, Trp138, Met142, TRP145, Thr149, VaL152, Glu156, Asn176, Asn179, Glu180, Leu183, Phe184, and Trp207 which forms a cylindrical or a channel shape in the EthR receptor. Fig. 5 shows intermolecular interactions between the docked ligands and the binding site residues. Some of the ligands formed hydrogen bond interactions along with π-staking and several hydrophobic interactions.

**Fig. 6:**
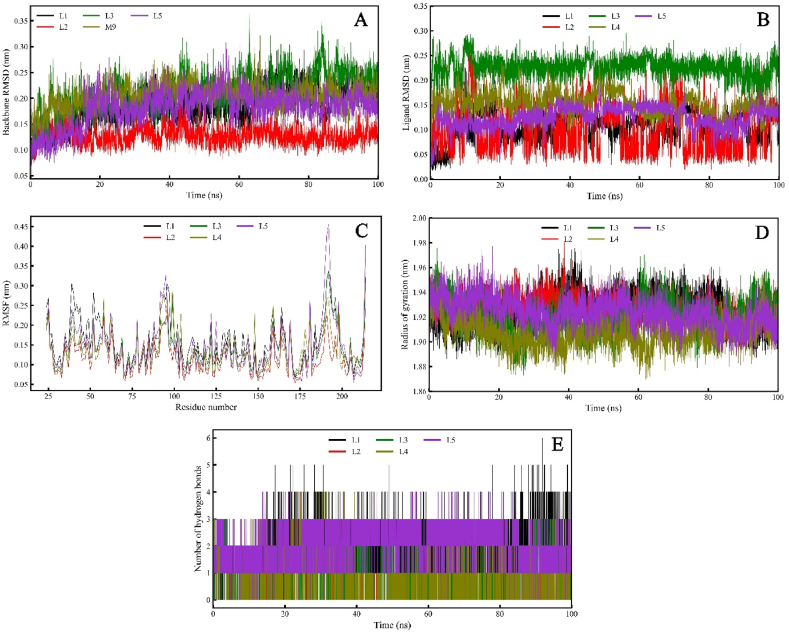
Molecular dynamics simulation of the docked complexes; (A) Protein backbone RMSD, (B) Ligand RMSD of docked ligands following molecular dynamics simulation analysis, (C) RMSF of residues from the EthR protein-ligand complex over 100 ns molecular dynamics simulation, (D) Radius of gyration for the EthR protein when bound to the docked ligands, (E) Hydrogen bond contacts between ligands and the protein during the 100ns MD simulation duration.

**Fig. 7:**
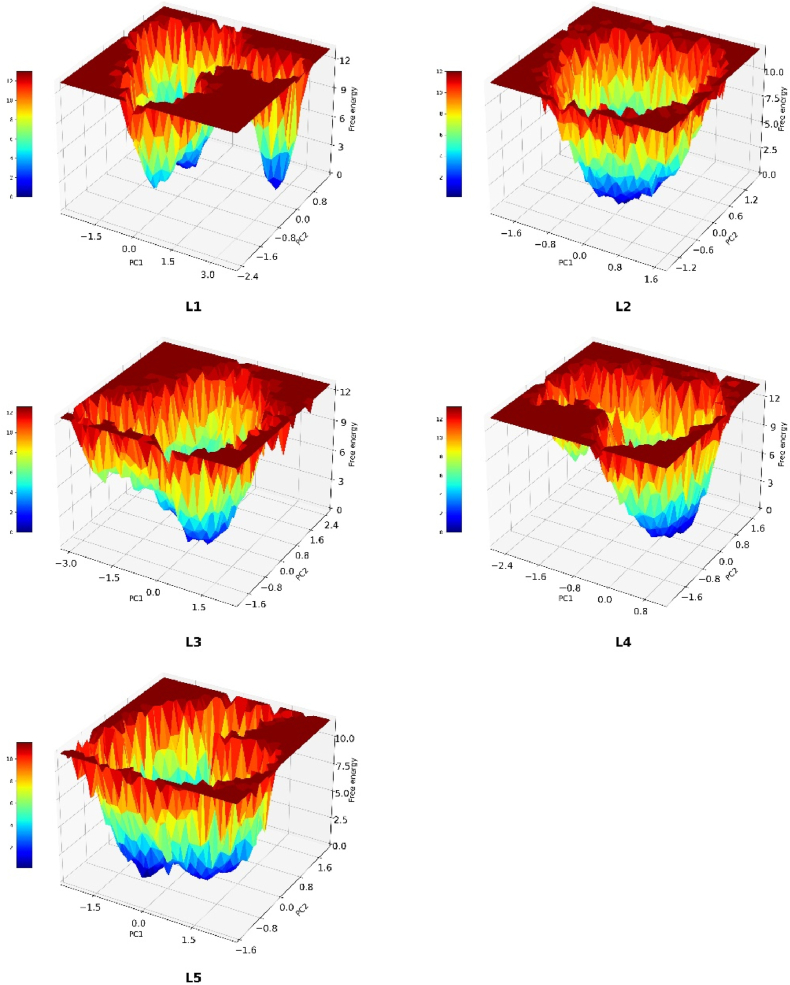
Free energy landscape for the protein-ligand interactions during the MD simulations following Principal Component Analysis (PCA) for the MD trajectories for the ligand-protein complexes of **L1** (Ethyl (2-amino-4-((4-fluorobenzyl)amino)phenyl)carbamate), **L2** (2-((2,2-Difluorobenzo [d] [1,3]dioxol-5-yl)amino)-2-oxoethyl (E)-3-(5-bromofuran-2-yl)acrylate), and **L3** (N-(2,3-Dihydrobenzo [b] [1,4]dioxin-6-yl)-4-(2-((4-fluorophenyl)amino)-2-oxoethoxy)-3-methoxybenzamide) were obtained as hit ligands from the Selleckchem database following molecular docking. **L4**, also known as Pretomanid ((*S*)-2-Nitro-6-((4-(trifluoromethoxy) benzyl)oxy)-6,7-dihydro-5H-imidazo [2,1-b] [1,3]oxazine) is potent antimycobacterial compound and **L5** (4-{3-[(phenylsulfonyl)amino] prop-1-yn-1-yl}-N-(3,3,3-trifluoropropyl)benzamide), is the co-crystallised ligand from the crystal structure of EthR (PDB: 4M3D).

After the ADMET studies, the final ligands were subjected to MD simulation for 100 ns with the explicit solvent model. These trajectories were analyzed to understand the stability of the complexes and as input for calculating the binding energy. The MD trajectory analysis of these complexes suggests several levels of knowledge about these selected compounds. The ligand RMSD shown in Fig. 6(B) points out the low RMSD for **L1**, **L2**, **L4** and **L5** below 0.20 nm, whereas the ligand L3 led to slightly higher RMSD of about 0.25 nm. In the case of protein RMSF analysis (Fig. 6(C)), all the complexes show a low RMSF except for the **L5**-EthR complex, which shows a spike in the RMSF in the ligand binding site, which lies between 175 and 200 residues and correlates with the protein RMSD (Fig. 6(A)). The radius of gyration for the ligand **L4**-EthR (Fig. 6(D)) is significantly distant from other complexes at 1.88 nm for several instances during the 100 ns MD simulation. The hydrogen bond formation and its retention are some of the exciting aspects of the stability of any protein-ligand complex. Fig. 6(E) shows hydrogen bond interaction between the molecules under study through the simulation. Ligands **L1**, **L3**, **L4** and **L5** show 2–3 hydrogen bonds with the receptor residues throughout MD simulation. The MD simulation trajectories were further used to analyse the free energy landscape for these ligands. The principal component analysis (PCA) for the MD trajectories was calculated. Two PCAs were plotted against the free energy as shown in Fig. 7, where the native ligand **L5** and docked ligand **L2** show the best results.

The docking calculations were validated by performing a self-docking experiment where we found an exceptionally low RMSD of 0.182 Å to the crystal structure, this process involved docking and redocking of the native ligand. This validated protocol was used for docking studies to obtain ligands with high dock scores and low RMSDs. The ligands obtained from ADMET filters, the physicochemical properties filter, and the docking calculations were subjected to MDS. The MDS provided insight into the binding of these ligands to the target protein. The ligands **L1**, **L2** and **L3** were the top molecules from the docking studies. These ligands showed hydrogen bond interactions with the key residues in the EthR binding site. The ligand **L1**, also known as retigabine, is a known anticonvulsant therapeutic; it was found to bind the EthR binding site by forming hydrogen bond interactions with Asn176 and Asn107, which are key residues. It also shows the highest dock score of −12.45 and binding energy of −36.68 kcal/mol calculated by the MM-GBSA method, which is higher than the standard TB drug Pretomanid and the co-crystallised ligand for this protein. The MDS results for **L1** suggest the formation of a stable complex with low fluctuations over the period of MDS.

The ligand **L2** also interacts with the EthR binding site with hydrogen bond interactions with Asn176 and Trp148, it also forms a stabilizing π-π interaction with the Phe110 and Trp103. **L2** docks with a score of −10.21 and binding energy of −32.57 kcal/mol. It also forms a stable complex with the binding site residues which is evident from its MDS-ligand RMSD which oscillates between 0.05 and 0.15 nm through the 100ns simulation. The free energy landscape (Fig. 7) for the **L2** shows a deep well with low free energy for this complex suggesting its stability. The ligand **L3** shows the second-highest dock score of −11.32 and a binding energy of −36.17 kcal/mol. Visual examination of the **L3**-EthR docked complex shows hydrogen bond interaction with the key residues Asn179 and Ala91 and several hydrophobic interactions with Trp145, Leu87, Trp207 and Trp103. The ligand RMSD for **L3** was observed to be very stable compared to the rest of the ligands as it fluctuated within a range of 0.05 nm (Fig. 6(B), green) and other parameters for this complex, like the protein RMSD, RMSF, and RoG, reflect the same stability. Furthermore, the hydrogen bonds formed are consistent through the 100 ns simulation, justifying its stable complex formation with the EthR. The standard drug molecule that came up from the set of 30 antiTb drug molecules was found to be **L4**, also known as Pretomanid, which is an antiTb prodrug molecule that is active against replicating and nonreplicating mycobacterium. **L4** binds with the EthR to form hydrogen bond interactions with the Asn179 and Tyr148, it forms the π-π interactions with Phe110 as in the case of **L2**. The dock score for the L4 is lowest at −9.31 and binding energy at −28.34 kcal/mol making it a weak binder compared to the newly found molecules. The MDS profile for **L4** was also found to be less stable than the rest of the ligands as it showed highly fluctuating ligand RMSD.

The co-crystallised ligand **L5** retained its crystallographic interactions with the residues Asn176, Asn179, Tyr148 and the π-π interactions with Phe110 and Trp103. It showed a docking score of −9.46 and a binding energy of −21.78 kcal/mol. L5 displayed a lower dock score and binding energy than the rest of the ligands. However, its MDS ligand RMSD shows a stable EthR interaction with RMSD between 0.05 nm and some fluctuations between 70 and 90 ns. This could result from its conformational exploration during the simulation as reflected in the RMSF plot. However, the hydrogen bond profile suggests stable interactions throughout the MDS. The free energy landscape (Fig. 7 (L1-L5)) also supports our findings; it shows a large area of low energy through the simulation (Fig. 7 L5), which is best compared to the rest of the ligands and obvious given it being the native bond ligand to this protein. The most exciting fact this study has highlighted is the structural features. L6 in Fig. 4 represents some common structural features, such as the fused ring systems, benzamide, and/or amide bonds, which are crucial structural features required in EthR inhibitors.

## 4 Conclusions

Tuberculosis has been a challenge to the world since prehistoric times, and with the advent of drug-resistant strains, it has become more challenging to treat this infection. The recent Covid pandemic, climate change, and socioeconomic and geopolitical conditions throughout the globe are driving us away from the solution for this curable infection. In this report, we have attempted and successfully identified three new molecules from the drug repurposing library that can be used for targeting EthR protein. These molecules were obtained after rigorous filtering of the database for their physicochemical, toxicological properties and safety, respectively. Compounds Ethyl (2-amino-4-((4-fluorobenzyl)amino)phenyl)carbamate) (**L1**), 2-((2,2-Difluorobenzo [d] [1,3]dioxol-5-yl)amino)-2-oxoethyl (E)-3-(5-bromofuran-2-yl)acrylate (**L2**), and N-(2,3-Dihydrobenzo [b] [1,4]dioxin-6-yl)-4-(2-((4-fluorophenyl)amino)-2-oxoethoxy)-3-methoxybenzamide (**L3**) are potential EthR inhibitor that would boost the activity of ETH in inhibiting the mycobacteria can help further improve the treatment of TB. All identified ligands are thought to be potential compounds that should be investigated for evaluating their biological activity to determine the inhibitory efficacy profile to aid in the anti-tubercular therapy targeting the EthR protein. Future therapeutic approaches may also be made possible by deriving of information on the identified hit compounds' activity and relationship for a better understanding of chemical space optimization. Furthermore, reaction kinetics investigations and/or the cellular thermal shift/melt assay can be used to assess the binding and unbinding pattern mechanism of suggested drug-like candidates for EthR inhibitors/modulators. The screened potential inhibitors/modulators for the EthR protein of Mtb could also pave for other therapeutic modalities in the future.

## Data availability

The data related to this work could be provided on request.

## CRediT authorship contribution statement

**Rupesh V. Chikhale:** Writing – review & editing, Writing – original draft, Supervision, Methodology, Formal analysis, Conceptualization. **Gaber E. Eldesoky:** Writing – original draft, Conceptualization. **Mahima Sudhir Kolpe:** Visualization, Validation, Formal analysis, Data curation. **Vikramsinh Sardarsinh Suryawanshi:** Investigation, Formal analysis, Data curation. **Pritee Chunarkar Patil:** Writing – original draft, Resources, Conceptualization. **Shovonlal Bhowmick:** Writing – review & editing, Writing – original draft, Methodology, Investigation, Formal analysis, Conceptualization.

## Declaration of competing interest

The authors declare the following financial interests/personal relationships which may be considered as potential competing interests:Gaber E. Eldesoky reports administrative support and statistical analysis were provided by Chemistry Department, College of Science, 10.13039/501100002383King Saud University, Riyadh 11451, Saudi Arabia. Dr Rupesh Chikhale is an associate editor to the Pharmaceutical Sciences Section of the Heliyon Journal. If there are other authors, they declare that they have no known competing financial interests or personal relationships that could have appeared to influence the work reported in this paper.
